# The impact of the French soda tax on prices and purchases. An ex post evaluation

**DOI:** 10.1371/journal.pone.0223196

**Published:** 2019-10-11

**Authors:** Sara Capacci, Olivier Allais, Celine Bonnet, Mario Mazzocchi

**Affiliations:** 1 Affiliation Department of Statistical Sciences, University of Bologna, Bologna, Italy; 2 Affiliation Institut National de la Recherche Agronomique (INRA), UR1303 ALISS, Ivry-sur-Seine, France; 3 Affiliation Toulouse School of Economics, University of Toulouse Capitole, Toulouse, France; SOAS, University of London, UNITED KINGDOM

## Abstract

We estimate the price and consumption effects of the 2012 French tax on sweetened non-alcoholic drinks using a difference-in-difference approach. Our identification strategy exploits Italian data as a natural control group. We use French and Italian Consumer Price Indices, purchase prices and quantities from the 2011 and 2012 Kantar and GfK home-scan surveys for two French regions and two neighbouring Italian regions, and expenditure data from the 2011 and 2012 Italian Expenditure Survey. We check for the robustness of our results by applying the difference-in-difference models using only French data and considering water as the benchmark (control) good. We find that the tax is transmitted to the prices of taxed drinks, with full transmission for soft drinks and partial transmission for fruit juices. The evidence on purchase responses is mixed and less robust, indicating at most a very small reduction in soft drink purchases (about half a litre per capita per year), an impact which would be consistent with the low tax rate. We find suggestive evidence of a larger response by the sub-sample of heavy purchasers. Fruit juices and water do not seem to have been affected by the tax.

## Introduction

Taxation of sweetened beverages as a mean to reduce the risk of excess weight and non-communicable diseases, especially in children, has been a key component of nutrition policies for many governments over the last decade. However, the ex post empirical evidence on the effectiveness of these taxes is still limited. In this study, we evaluate the impact of a tax on sweetened non-alcoholic drinks introduced in France in January 2012, and we provide quasi-experimental evidence on its effect on prices and purchased quantities.

Taxation of soft drinks dates back to 1933, when California introduced a 7% sales tax. By the end of 2018, 34 states and seven cities in the United States were taxing soft drinks [1], and some city-level taxes were adopted following popular ballots [2]. Outside the US, with some exceptions (e.g. Scandinavian countries), the implementation of the so-called soda taxes has spread only in recent years. According to the Nourishing data-base regularly updated by the World Cancer Research Fund, 31 national governments have enacted soda taxes between 2011 and 2019, including an 18% tax on sugary drinks introduced in Chile in 2015 [3] and a $ 0.07 per litre tax in Mexico [4].

In Europe, taxes on soft drinks are currently implemented in the UK (from 2018 there is an industry levy on sugary drinks up to £0.24 per litre depending on their sugar content), Ireland (from 2018, up to €0.30 per litre), Belgium (from 2016, €0.068 per litre), Hungary (from 2011, $ 0.24 per litre), Norway (from 1981, €0.35 per litre), Portugal (from 2017, up to €0.16 per litre), Spain (Catalunya in 2017, up to 0.12 €per litre) and Finland (excise duty tax introduced in 1940, currently up to €0.22 per litre, again depending on sugar content).

The French soda tax was introduced in January 2012 and set to €0.0716 per litre. It applies to all sweetened drinks, including sugar substitutes used in diet drinks, and is paid by manufacturers, processors and importers.

Despite the growing spread of this type of fiscal measures across the world, there are conflicting visions about their effectiveness in reducing consumption of sugary drinks and improve public health. According to the current evidence, the main outcome has been the generation of revenues rather than significant changes in consumption behaviors. This has been explained with the relatively low level of the taxes [5–7]. Based on the existing evidence, a report published by the World Health Organization in 2016 [8] suggests that these taxes should raise the price of targeted drinks of at least 20% to generate meaningful impacts in terms of calorie intakes, weight and risk of non-communicable disease.

The evidence base is still incomplete, and the findings inconsistent, not least because studies are heterogeneous in terms of design, tax levels and aims. Until recently, most of the quantitative evidence has been based on demand simulations. These simulations necessarily rely on key empirical assumptions on the pass-through from producer prices to retail prices, and rest on elasticities and behavioural parameters whose estimates depend heavily on the demand model specification [9] and the variability in price data relative to the tax level.

The rising adoption of soda taxes in recent years, as well as the increasing availability of purchase data, should allow a more accurate ex post assessment of their effects, at least in the short-term (see for example the systematic review by Redondo et al. [10]). Recent ex post evaluations are suggestive of soda taxes generating significant reduction in purchased quantities. Colchero et al. [4] exploit panel data on food and drink purchases of 6,645 Mexican households to estimate an average reduction of 7.6% in purchased volumes of taxed beverages in Mexico over the first two years of the tax implementation. The 2014 Mexican tax amounted to 1 peso per litre (about 0.08 USD at that time), and a previous study [11] had shown a full pass-through to consumer prices. Two studies have explored the effects of the Chilean soda tax, with estimates of the reduction in purchases of the 18% taxed soft drinks which ranges from 3.4% [12] to 21% [13], despite both studies finding a low pass-through.

The ex post evidence gathered from the city-level Berkeley soda tax is particularly interesting for two reasons. First, the level of the tax ($ 0.34 per litre) is much higher than most of the fiscal measures adopted elsewhere. Second, the tax was adopted in November 2014 as a ballot measure, and the debate prior to the vote is likely to have generated information effects on consumption beyond the mere price effect. Two studies [14, 15] have evaluated the impact of the tax exploiting neighbouring areas as control groups. As for the other ex post studies, there was robust evidence of a pass-through to retail prices. The estimates also show a large reduction in purchased quantities of taxed drinks over the first year of implementation, estimated at -9.6% by Silver et al. [15] and at 21% by Falbe et al. [14], although both studies also register an increase in purchases in the neighbouring (control) areas, 6.9% and 4%, respectively. The Philadelphia city-level tax has been also evaluated ex-post using a difference-in-difference approach relative to control areas not subject to the tax. The empirical evidence is that the $ 0.44 per litre tax had a full pass-through and generated a significant reduction in household purchases, estimated at 0.26 litres per shopping trip [16]. This substantial reduction is confirmed by another retail-level study which found that overall sales of taxed drinks fell by 51% following the implementation of the tax [17].

To the best of our knowledge, only two studies conduct an ex post evaluation of the French soda tax focusing on the effect of the excise tax on retail prices [18, 19], and there are no studies looking at the ultimate impact on purchases or consumption. Based on a large dataset on retail prices, Berardi et al. [18] consider a sub-set of non-taxed goods with pre-tax price patterns similar to the taxed categories as a natural control group. Their soda category, which includes regular and diet sodas, exhibits an average pass-through around 7 euro-cents per litre over the first 6 months of the tax. Fruit drinks and flavored waters show a slightly smaller pass-through. These empirical findings are consistent with a simulation-based study by Bonnet and Réquillart [20] based on pre-tax data, that predicted that French firms would be likely to transmit, and even over-transmit, cost changes or excise taxes to consumers. Etilé et al. [19] estimate the impact of the tax on Exact Price Indices of sweetened beverages (considering beverages containing both sugar and sweeteners). Their identification strategies rest on a before-after approach and a difference-in-difference (DID) design using water as a control good. They find that the soda tax significantly affected the Exact Price Indices of sweetened beverages (+4%), corresponding to an overall pass-through of about 39%.

In this study, we evaluate the impact of the French soda tax on retail prices and purchased quantities. We also explore whether the tax has had differential effects on households with a heavier consumption of taxed drinks. Our evaluation is based on panel household purchase data collected through home-scan devices in four regions in the twelve months preceding and following the introduction of the tax. We consider two French regions (Rhone Alpes and Provence-Alpes-Cote d’Azure) where households are exposed to the tax, and two neighbouring Italian regions (Piemonte-Val D’Aosta and Liguria) that act as a natural control group. Because of potential structural differences among these regions, we adopt a DID panel regression to control for selection on non-observable variables, allowing for fixed cross-section effects and country-specific linear time trends. We check for the robustness of our results by adopting different specifications of the DID model, and by considering alternative sources of data, such as the official Consumer Price Indices (CPIs) at national level, and household purchases for the Italian Household Expenditure Survey as an alternative to the home-scan dataset.

## Policy background

The French tax on sweetened soft drinks was incorporated in the 2012 French budget bill (Law No. 2011-1977) and entered into force on January 2012. It applies to all non-alcoholic beverages containing added sugar (e.g. sodas, fruit juice) or sweeteners (e.g. diet drinks) and amounts to 7.16 eurocents per litre excluding VAT, or 7.55 eurocents per litre at the retail level where a 5.5% VAT is applied. The tax is paid by manufacturers and processors in France and by French importers.

In its initial proposal within the Draft Budget 2012, the tax was lower (3.58 cents per litre), it did not apply to artificially sweetened drinks and it was framed within the broader scope of the French National Nutrition and Health Program among public measures targeting eating patterns to promote healthier lifestyles. The explicitly stated rationale of the tax was originally to discourage the consumption of sugary and sweetened beverages and direct consumers towards other beverages.

The proposal caused a strong opposition by the French Food Industry Association and by those producers holding the largest shares in the non-alcoholic beverage market [21]. The reference to the National Nutrition and Health Program and to healthy eating objectives does not appear in the final text of the law, approved on December 2011, where the tax level is doubled relative to the original proposal.

## Data sources

Our analysis is based on three different data sources: (a) commercial home-scan panel (HSP) data from Kantar WorldPanel France and GfK Italy; (b) household purchase data from the Italian Household Expenditure Survey (HES); (c) official Consumer Price Indices (CPIs) at the national level.

The home-scan dataset provides household level longitudinal information on purchases for home consumption and on purchase prices. Since the available HSP for the two Italian regions is relatively small and less precise than its French counterpart, we use data from the Italian HES to assess whether noise in the data may affect impact estimates. Estimates on the tax transmission to prices are also based on official CPIs, which are available at the national level for both countries, but with a higher level of aggregation relative to the home scan data.

### Home-scan panel data

We use HSP data provided by Kantar WorldPanel France and GfK Italy from a random sample of French and Italian households living in four neighbouring regions: Rhone Alpes and Provence-Alpes-Cote d’Azure in France and Piemonte-Val D’Aosta and Liguria in Italy.

The harmonized dataset consists of 2,928 French households and 400 Italian households observed over the period between 1 January 2011 and 31 December 2012, conditional on at least one purchase of non-alcoholic beverages in each of the two years. Weekly expenditures and purchased quantities are available for the following drink categories: regular soft-drinks; diet soft-drinks; non-pure fruit juices; mineral water; pure fruit juices. All drinks included in the first three categories are subject to the tax. The regular soft drink category includes flavoured mineral waters, also taxed, whereas the mineral water category only includes non-taxed products. Pure fruit juices with no added sugars are also exempt from the tax. By taking the ratios between expenditures and purchase quantities, we obtain the unit prices paid by the household for each drink group.

In addition, the harmonized dataset includes a set of household characteristics: household size, presence of children aged under 15, age of the person responsible for food purchases and a binary variable for job status (employed or unemployed). Harmonization of information on household incomes is not possible as income information was not collected in the Italian survey. As explained in the Appendix, we adopt a classification rule to achieve some comparability in terms of socio-economic status and explore the role of inequalities.

### Italian household expenditure survey

The available sample for the two Italian regions from the Italian HSP provides longitudinal information on 400 households that have purchased at least one non-alcoholic drink in each of the two years. The Italian HSP sample is much smaller than its French counterpart, hence affected by a larger sampling error, and more sensitive to outliers and measurement errors. Thus, we extract data from the 2011 and 2012 Italian HES for the same regions, with the aim of assessing the robustness of impact estimates based on home scan data. The Italian HES runs on a yearly basis and collects information on purchase expenditures from about 23,000 Italian households. Relative to the home-scan data, this dataset has several shortcomings: (1) there is no longitudinal dimension as a new sample is extracted every year; (2) expenditures for each household are recorded through a two-weeks diary compared to the continuous monitoring of the home scan panel; (3) there is no information on purchased quantities, only monetary values of expenditures; and (4) there is a lower level of detail (i.e. purchase data refer to soft drinks, fruit juices and water). However, the number of households in the relevant Italian regions (Liguria, Piemonte and Val D’Aosta) is relatively large (3,136 households in 2011, 3,243 in 2012) compared to the 400 households available in the home-scan panel dataset, and the probabilistic design of the survey ensures the representativeness of the data in terms of demographic characteristics.

As the HES records expenditures without providing information on purchased quantities, the latter were estimated using purchase price information obtained from the home scan data-set. More specifically, the HES provides information on the month each household entered the survey. Purchase quantities were estimated by dividing the household expenditure values by the average price for the same category, month and region as computed from the Italian HSP.

### Consumer price indices

We use official monthly national CPIs from 2007 to 2016 as released by INSEE for France and ISTAT for Italy to investigate the extent of the pass-through of the tax to retail prices. National CPIs from both countries are available for the following drink categories as the highest level of product detail: soft drinks (including both sweetened and diet beverages); mineral and spring waters; fruit and vegetable juices. In order to account for the potential effects of differential inflation rates (which in 2012 was +3% in Italy and +1.3% in Metropolitan France), these indices were deflated by an all-item consumer price from the same sources.

## Descriptive statistics

### Demographics

Table 1 shows the average sample characteristics for the two HSPs, and for the Italian HES. The French HSP sample has a larger proportion of households with children aged less than 15, a higher proportion of active (employed) HRPs. The percentage of households with a medium-high and high socio-economic status is higher in the two Italian regions relative to their French counterparts. Since the classification of socio-economic status is relative (country-specific), this comparison suggests that the two Italian regions are wealthier than the French ones relative to the respective national benchmark. The two samples also differ in terms of age distribution, as the Italian sample has a lower proportion of young households and a higher proportion of households where the person responsible for food shopping is over 55. These socio-demographic differences between France and Italy emerge from both the Italian HSP and the Istat HES, although with different intensity. The information in Table 1 suggests a systematic difference in the demographic structure between the French and Italian regions.

**Table 1:** Descriptive statistics: Household demographics, by country and data source.

	French Regions	Italian Regions	
		HPS^a^	HES^b^
Presence of children < 15y.o.	0.333	0.230	0.180
	(0.471)	(0.421)	(0.384)
RP^c^ employed	0.714	0.412	0.462
	(0.452)	(0.493)	(0.453)
Low SES^d^	0.287	0.183	0.150
	(0.452)	(0.386)	(0.357)
Medium-low SES	0.124	0.212	0.200
	(0.330)	(0.409)	(0.400)
Middle SES	0.416	0.297	0.300
	(0.493)	(0.457)	(0.458)
Medium-high SES	0.086	0.193	0.200
	(0.281)	(0.395)	(0.400)
High SES	0.087	0.115	0.150
	(0.281)	(0.319)	(0.357)
RP<35 years old	0.221	0.08	0.077
	(0.415)	(0.271)	(0.266)
RP 35-44 year sold	0.248	0.223	0.159
	(0.432)	(0.416)	(0.366)
RP 45-54 years old	0.184	0.215	0.199
	(0.388)	(0.411)	(0.399)
RP 55-64 year sold	0.163	0.21	0.186
	(0.370)	(0.408)	(0.389)
RP >64 year sold	0.183	0.273	0.380
	(0.386)	(0.446)	(0.485)
Household size	2.516	2.547	2.149
	(1.143)	(1.053)	(1.077)
Number of households	2928	400	6379
Liguria	-	82	2012
Piemonte-Val D’Aosta	-	318	4367
Provence-Alpes-Cote d’Azur	1225	-	
Rhone Alpes	1703	-	

Numbers in brackets are Standard Deviations

^(a)^ Italian Home Scan Panel (HSP)

^(b)^ Italian Household Expenditure Survey (HES)

^(c)^ Household Reference Person (RP)

^(d)^ Socio-Economic Status (SES) is classified as: bottom 15%(low); 15th-35th percentile (medium-low); 35th-65th percentile (middle); 65th-85th percentile (medium-high); top 15% (high). See the Appendix for details.

Furthermore, figures from the HES survey indicate larger proportion for elderly households, less children under 15 and a relatively higher wealth compared to the HSP. These differences are consistent with a higher difficulty in recruiting elderly households in HSPs. Unfortunately, household weights for the home scan data-sets were not available.

### Prices

The first condition for a tax to influence consumption is obviously that it is transmitted to consumers. We exploit official CPIs and commercial home-scan data to test whether the policy has actually changed the costs borne by the households to purchase unhealthy drinks. In principle, producers and/or retailers might fully or partially absorb the excise tax, leading to incomplete transmission.

**Fig 1:**
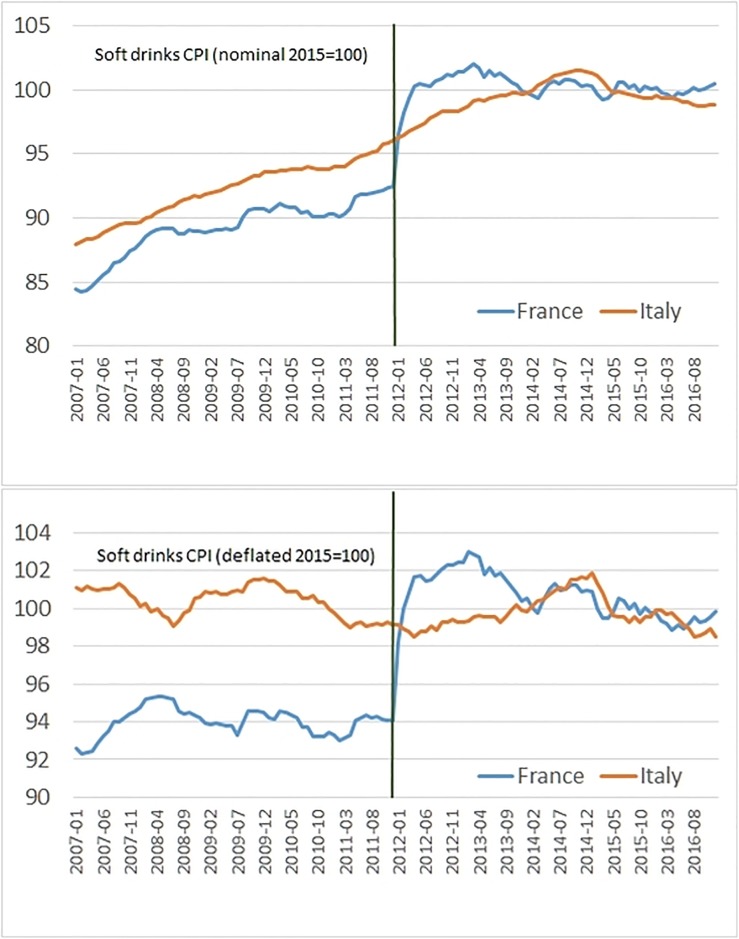
Nominal and real soft drink consumer price indices in France and Italy, 2007-2016 (2015 = 100).

Fig 1 displays nominal and deflated CPIs patterns from 2007 to 2016. The graph is suggestive of a meaningful real price increase of soft drinks in France in the first two years of the tax implementation, while it shows a substantial overlap of French and Italian price patterns from 2014.

The ratio between expenditures and purchased quantities as obtained from the HSP determines the purchase unit values, that are a combination of the retail price levels and consumer choices in terms of quality and basket composition. There is a consolidated literature on the difference between unit values and prices [22, 23], where the elicitation of retail prices rests on the assumption that households living in the same geographic area in a given time period face the same price, and any heterogeneity observed at that level stems from different household choices rather than different prices. Thus, a simple but effective way to obtain indirect estimate of retail prices consists in computing the weekly averages of unit values across households living in the same region.

Table 2 shows the averages of the monthly CPI (2015 = 100) and of the weekly purchase prices in Euros for France and Italy, in 2011 and 2012. The CPIs are at the national level and purchase prices refer to the regions within each country available in our home-scan dataset. All figures are in real terms as the price series were deflated by the all-item CPI of the respective country. The table also reports the percent change between 2011 and 2012 as estimated by a basic pre-post model on the natural logarithms of the price series.

**Table 2:** Average prices and pre-post differences.

	France			Italy		
	2011	2012	Difference^a^	2011	2012	Difference^a^
	**Real CPI (2015 = 100)**					
Soft drinks	93.84	101.36	0.077***	99.23	98.99	-0.002**
	(0.50)	(1.20)	(0.002)	(0.21)	(0.28)	(0.001)
Fruit Juices	101.03	104.70	0.036***	101.77	101.59	-0.002
	(2.10)	(0.54)	(0.004)	(0.43)	(0.28)	(0.001)
Water	108.61	107.47	-0.011***	103.27	101.42	-0.018***
	(0.67)	(1.08)	(0.003)	(0.57)	(0.48)	(0.001)
	**Real purchase prices from home-scan data (€/litre)**					
Soft drinks	1.08	1.14	0.056***	1.10	1.12	0.012
	(0.06)	(0.06)	(0.007)	(0.18)	(0.18)	(0.022)
Regular soft drinks	1.12	1.18	0.057***	1.13	1.17	0.039**
	(0.07)	(0.08)	(0.008)	(0.17)	(0.16)	(0.018)
Diet soft drinks	0.95	1.02	0.067***	0.94	0.83	-0.150**
	(0.05)	(0.06)	(0.007)	(0.43)	(0.37)	(0.070)
Fruit Juices	1.46	1.49	0.025***	1.51	1.38	-0.075***
	(0.04)	(0.04)	(0.004)	(0.40)	(0.25)	(0.028)
Non-pure fruit juices	1.62	1.65	0.018***	1.41	1.38	-0.026
	(0.05)	(0.04)	(0.004)	(0.38)	(0.35)	(0.034)
Pure fruit juices	1.31	1.33	0.021***	1.56	1.36	-0.118***
	(0.06)	(0.07)	(0.006)	(0.50)	(0.30)	(0.034)
Water	0.41	0.41	-0.002	0.26	0.26	-0.014
	(0.02)	(0.02)	(0.006)	(0.04)	(0.04)	(0.019)

^(a)^ Pre-post model in logs of real prices. CPIs monthly national series are from INSEE and ISTAT for France and Italy, respectively. Home-scan purchase prices are deflated by national overall CPIs.

* p<0.1;

** p<0.05;

*** p<0.01.

The data provides consistent evidence of an increase in all prices but those of water in France, whereas all Italian prices where stable or decreasing, with the exception of regular soft drinks. The rough difference in soft drink prices in France as captured by the real CPIs is around 7.7%. Considering purchase prices from home-scan data, the average price of soft drinks went up by about 6 eurocents, or 5.7%. The estimated price increase is similar for regular soft drinks and diet soft drinks, and smaller for fruit juices (3 eurocents or +2.5%), again with a negligible difference between non-pure juices (taxed) and pure fruit juices (not taxed). Considering the Italian price data, the analysis of CPIs suggests stable prices, and a small reduction for water (-1.8%). Soft drinks purchase prices for the Italian regions are very similar to the French ones in 2011, but the average increase in 2012 is smaller and non-significant (2 eurocents, or +1.2%). However, this average change is the outcome of a significant increase in the price of regular soft drinks (+3.9%) and a relatively large decline in the real price of diet soft drinks (-15%), although these estimates may also depend on the smaller sample size and larger variability in the Italian home-scan data, as reflected by the larger standard errors. Interestingly, real prices of mineral and spring water are stable in both country, with an average price which is substantially larger in France (0.41 vs. 0.26 euros per litre).

### Purchased quantities

Table 3 shows average per-capita drink purchases in 2011 and 2012 in the Italian and French sub-samples as estimated from the HSP data. On average, total beverage purchases in the Italian regions are twice as large as those in French regions, mainly because of mineral water consumption, which is particularly large in Italy (around 3.3 litres per-capita per week in 2011 compared to 1.1 litres per capita in the French regions). With regard to taxed beverages, the largest difference is observed for regular soft drinks. On average, consumers in the French regions purchased about one can per week in 2011, whereas the Italian region counterparts purchased almost two cans per week. However, purchases of diet sodas and non-pure juice were slightly lower in the Italian regions.

**Table 3:** Average purchased quantities by country and year (weekly per capita litres).

	French Regions			Italian Regions		
	2011	2012	Difference	2011	2012	Difference
Taxed drinks	0.615	0.605	-0.010	0.853	0.741	-0.112**
	(0.767)	(0.763)	(0.020)	(0.651)	(0.653)	(0.046)
Regular soft drinks	0.337	0.326	-0.011	0.62	0.537	-0.083**
	(0.576)	(0.539)	(0.015)	(0.549)	(0.520)	(0.038)
Diet soft drinks	0.111	0.114	0.003	0.085	0.064	-0.021*
	(0.364)	(0.382)	(0.010)	(0.196)	(0.159)	(0.013)
Non-pure juice	0.167	0.164	-0.003	0.148	0.14	-0.008
	(0.247)	(0.276)	(0.007)	(0.166)	(0.220)	(0.014)
Non-taxed drinks	1.278	1.349	0.071	3.325	3.403	0.079
	(1.690)	(1.824)	(0.046)	(2.584)	(2.786)	(0.190)
Pure juice	0.175	0.189	0.014*	0.036	0.026	-0.010*
	(0.273)	(0.289)	(0.007)	(0.077)	(0.073)	(0.005)
Water	1.102	1.159	0.057	3.289	3.377	0.088
	(1.636)	(1.761)	(0.044)	(2.574)	(2.770)	(0.189)
Total drinks	1.896	1.957	0.067	4.209	4.169	-0.078
	(1.987)	(2.106)	(0.055)	(2.85)	(3.06)	(0.214)
Number of obs.	2958	2958		400	400	

* p<0.1;

** p<0.05;

*** p<0.01.

The pre-post comparison of national averages shows a reduction of around 10 ml in per capita weekly purchases of taxed drinks after the introduction of the tax in French regions, a value which is very small and non-significant. As a matter of fact, there is no significant changes in purchased quantities between 2011 and 2012 in France, with the exception of a small increase in purchases of pure fruit juice (+14 ml per capita per week). Instead, we observe a relatively larger and significant reduction in soft drink purchases in Italy, where no tax is enforced. While these figures are already suggestive of a lack of impact of the French soda tax, the question is whether a rough double difference computation is able to capture the potential effects of the tax, since it does not account for the panel structure of the data, hence for any heterogeneity across households (e.g. heavy consumers vs. occasional purchasers) and over time (e.g. pre-existing differential trends, seasonal effects). Given the relatively small sample size of the Italian HSP, a comparison with the data from the Italian HES is provided in Table 4, which shows average purchase quantities as measured by the HSP and estimated HES quantities obtained from dividing HES household expenditure by the estimated average prices from the HSP. The average reduction in purchases of soft drinks captured by the HSP disappears when considering HES data. Estimates of per capita purchases are also lower in the HES in both years. This systematic difference is consistent with the older and wealthier HES sample relative to the HPS, but might also reflect some underestimation associated with the bi-weekly diary measurement of the HES compared to continuous home scan monitoring in the HPS. However, purchases of fruit juices and water are higher in the HES, which means that these differences are more likely to relate to the sample characteristics than underestimation. The fact that the HPS captures a significant change in soft drinks, whereas the HES reports significant changes in fruit juice and water purchases suggests that measurement errors may be important relative to actual changes in purchasing behaviours. It is not straightforward to say which survey has a higher measurement quality, but comparing impact estimates using the two different surveys enables us to assess their sensitiveness to noise in the data.

**Table 4:** Average purchased quantities in Italy, by data source (weekly per capita litres).

	Italian Home-scan data			Italian HES^a^		
	2011	2012	Difference	2011	2012	Difference
Soft drinks	0.705	0.601	-0.104**	0.548	0.526	-0.022
	(0.596)	(0.557)	(0.041)	(1.318)	(1.369)	(0.034)
Fruit Juices	0.184	0.166	-0.018	0.390	0.436	0.046*
	(0.196)	(0.239)	(0.015)	(0.930)	(1.159)	(0.026)
Water	3.289	3.377	0.088	5.598	5.104	-0.495**
	(2.574)	(2.770)	(0.189)	(8.996)	(8.929)	(0.224)
Number of obs.	400	400		3136	3243	

Standard Deviations and Standard Errors in brackets.

^(a)^ Italian Household Expenditure Survey (HES).

* p<0.1;

** p<0.05;

*** p<0.01.

## Methodology

### Difference-in-difference model

In order to estimate the impact of the soda tax on beverage prices and purchases we consider the two neighbouring Italian regions as a reasonable control group for the French regions under analysis, and resort to a DID framework. Similarly, the DID framework can be applied to national CPIs for France and Italy. Our identification strategy rests on the assumption that any border effect could be ignored. This assumption is reasonably safe even when we consider regional data, given the dimensions of the four regions, as only a very small number of households in our sample is located at a distance which makes the cross-border trip convenient (for example, someone living in Nice should drive about 25 miles to cross the border, and pay about €2.50, which correspond to the total amount of the tax for the purchase of about 33 litres of taxed drinks). Thus, we exploit the panel structure of the HSP data and estimate a set of DID models which allow for fixed cross-sectional effects and differential time trends. The specification of the general model is the following:

$$\begin{array}{c} Y_{ht}=\gamma_{h}+\lambda_{0}Trend_{t}+\lambda_{1}F_{h}\cdot Trend_{t}+\zeta_{hs}+\delta T_{ht}+\eta_{ht} \end{array}$$ (1)

where *Y*_*ht*_ is the outcome observed on cross-sectional unit *h* at time *t*; *γ*_*h*_ are cross-sectional fixed effects; λ_0_*Trend*_*t*_ + λ_1_*F*_*h*_ ⋅ *Trend*_*t*_ are linear time trends, allowing for a different slope between French observations (*F*_*h*_ = 1) and Italian observations (*F*_*h*_ = 0); *ζ*_*hs*_ is a set of quarterly dummies to capture seasonal effects; *T*_*ht*_ is the DID interaction term, which assumes a value of 1 for French observations in 2012, and 0 otherwise and *η*_*ht*_ is a randomly distributed error term. Under the DID approach, the coefficient *δ* yields the average effect of the tax on exposed observations.

To explore the average effect of the tax on prices we estimate Eq (1) on national CPIs as provided by the National Statistical Offices and on regional average prices as computed using home-scan data. To explore the average effect of the tax on purchases we estimate Eq (1) on household-level purchased quantities. We use home-scan data for the two French regions, and we provide results using either HPS and HES data for the two Italian regions as the natural control group.

According to the outcome variable explored, cross-sectional fixed effects have different specifications which depend on the unit of analysis *h* (i.e. country, region or household) and the nature of the data-set, as the HES data are not longitudinal.

### Outcome variables

#### National consumer price indices

A first estimate of the pass-through of the tax to consumer prices is based on official national CPIs. The DID model in Eq (1) is estimated using French and Italian monthly time series, and the specification of the fixed cross-sectional effects reduces to two national intercepts. The model is estimated for each of the three drink categories for which data are available, i.e. soft drinks (regular and diet), fruit juices (pure and non-pure), and water. The price indices are taken in natural logarithms, so that the estimated treatment effect can be interpreted as a percent change.

#### Regional average prices

In order to explore the pass-through of the tax at the higher level of item disaggregation provided by the home-scan data, the DID Eq (1) is estimated on weekly average regional prices computed on home-scan data. For each of the four regions, the price of each drink category is computed as the average of the unit values paid by each household for those drinks in a given week. While averaging unit values does not rule out that some of the price variation across regions and weeks might also depend on aggregation and quality choices, allowing for fixed regional effects and quarterly time effects controls for these potential sources of heterogeneity. Hence, in our DID price model, the resulting dataset consists of a panel of four cross-sectional units (the regions) and 104 time periods (one for each week over the two years of our data), and the model specification includes four regional fixed effects. As for price indices, the outcome variable enters Eq (1) in natural logarithms.

#### Household average purchases

Eq (1) is estimated on the household-level HSP data where purchased quantities are aggregated for each household over a period of 13 weeks (one quarter). The aggregation over a quarter mitigates the potential “zero bias” associated with stockpiling and heterogeneity in purchase frequencies, but we refer to the average weekly per capita purchase as a measurement unit, for ease of interpretation. In the HSP case, the estimation dataset is a balanced panel that includes all households in each of the eight quarters of 2011 and 2012, including zeroes when the household has not purchased the product in that quarter. The model specification includes household-specific fixed effects.

Eq (1) is also estimated using HSP data for France and HES data for Italy. In this case, the data cannot be treated as longitudinal, given the cross-sectional nature of the HES survey. Thus, we treat all French HSP quarterly observations as independent cross-sectional observations, and we only consider regional fixed effects.

#### Purchases by heavy consumers

Considering the possibility of heterogeneous effects of the tax, we estimate Eq (1) on the sub-sample of heavy consumers, defined as those in the top 25% in terms of their annual per-capita household purchases of each category in the year 2011, before the tax was introduced. For example, when considering regular soft drinks and HPS data, the top 25% corresponds to an average yearly purchase of 20.9 litres and 40.9 litres in France and Italy, respectively. When using the Italian HES data, heavy consumers in the top quartile are those purchasing more than 30.5 litres of soft drinks.

## Results

### Impact on prices

Table 5 shows estimates of the tax effects on national and regional consumer prices using Eq (1) for the two data-sources, national CPIs and weekly regional prices obtained from the HSP dataset.

**Table 5:** Tax impact on real prices.

	Weekly regional prices	Monthly CPIs	
	2011-12	2007-16	2011-12
Soft drinks	0.047*	0.082***	0.056***
	(0.018)	(0.003)	(0.005)
Regular soft drinks	0.049*	-	-
	(0.019)		
Diet soft drinks	0.139	-	-
	(0.068)		
Fruit Juices	-0.013	0.042***	-0.002
	(0.044)	(0.006)	(0.009)
Non-pure fruit juices	0.006	-	-
	(0.031)		
Pure fruit juices	-0.000	-	-
	(0.019)		
Water	-0.009	-0.006	0.001
	(0.013)	(0.006)	(0.007)
N	208	240	48

Prices are real (deflated by the overall CPI) and in logs.

* p<0.1;

** p<0.05;

*** p<0.01.

Considering only the window between 2011 and 2012, the estimated effect of the tax on CPIs for soft drinks is a 5.6% increase, and the corresponding estimate using HSP weekly regional prices s 4.7%. The HSP data allow to estimate specific tax effects on regular soft drinks (+4.9%) and diet soft drinks (+13.9%, but not significant). The tax has no significant effects on the aggregate fruit juice category or water. When the time window is expanded to include 5 years before and after the tax introduction, the application of the difference-in-difference model to CPI data returns a larger impact on soft drink prices (+8.2%) and there is evidence of significant transmission to fruit juice prices (+4.2%).

The estimation of a higher transmission when using CPIs relative to average purchase prices is in line with expectation, as the former are based on shelf-price data collection and a fixed basket composition, whereas the latter depend on the basket composition of goods that have been actually purchased, which evolves over time. In presence of price increases, consumers may rearrange their basket and move towards cheaper good, an adjustment which is captured by the HSP regional weekly prices, but not by the CPIs.

Considering that the average 2011 price for soft drinks in France was 1.08 €/litre (estimated using the home-scan data, see Table 2), a 4.7% real increase corresponds to about 5 eurocents per litre, and an 8.2% increase to 8.9 eurocents/litre. This, compared to the (nominal) excise tax of 7.55 eurocents per litre, shows that the tax transmission to soft drink prices is likely to have been complete, in the most conservative scenario at least 66% of the tax has been transmitted to retail prices, and our estimates are even suggestive of a potential over transmission, as envisaged by previous studies [20].

### Impact on purchased quantities

Table 6 reports estimates of the tax impact on purchased quantities, as obtained by applying Eq (1) to the HSP data for both countries, and using HES data for Italy. The small change in purchases of soft drinks is negative, but it emerges as non-significant with both Italian HSP and HES data. The only significant estimates refer to changes in purchases of fruit juices or water with HSP data, which are not confirmed when the counterfactual data are taken from the Italian HES. Overall, these results suggest that no clear effect of the tax can be detected, at least during the first year of application.

**Table 6:** Tax impact on purchased quantities, whole sample.

	French and Italian HSP^a^ data	French home scan data and Italian HES^b^ data
Soft drinks	-0.025	-0.112
	(0.039)	(0.057)
Regular soft drinks	-0.017	-
	(0.035)	
Diet soft drinks	-0.008	-
	(0.015)	
Fruit Juices	0.088***	-0.039
	(0.017)	(0.018)
Non-pure fruit juices	0.045***	-
	(0.014)	
Pure fruit juices	0.044***	-
	(0.009)	
Water	-0.682***	0.303
	(0.187)	(0.144)

^(a)^ Home Scan Panel (HSP)

^(b)^ Italian Household Expenditure Survey (HES)

Dependent variables are per capita purchased quantities.

Standard errors (in brackets) are clustered by household when using HSP data.

* p<0.1;

** p<0.05;

*** p<0.01.

Table 7 applies the same models to the sub-set of heavy purchasers, intended as those in the top quartile in terms of purchases of each good before the introduction of the tax. The estimated effects are now larger, the application to HSP data suggests a 132 ml reduction per capita per week in purchases, more than half a litre per month. While the standard error of this estimate is large, estimates using HES data for Italy are even larger and significant. Results on fruit juice are inconsistent. With HSP data, the reduction in soft drink purchases is compensated by a corresponding increase in juice (pure and non-pure), but the model applied to the Italian HES data estimates a significant (183 ml) decrease. Estimates for water are both large and negative, but non-significant. Overall, the application to the sub-group of heavy consumers are suggestive of some tax effects, at least on soft drinks, but estimates are more sensitive to noise in the data due to the smaller sample sizes.

**Table 7:** Tax impact on purchased quantities, heavy purchasers.

	French and Italian HSP^a^ data	French home scan data and Italian HES^b^ data
Soft drinks	-0.132	-0.599**
	(0.123)	(0.109)
Regular soft drinks	-0.098	
	(0.107)	
Diet soft drinks	-0.058	
	(0.055)	
Fruit Juices	0.165***	-0.183*
	(0.056)	(0.059)
Non-pure fruit juices	0.103**	
	(0.046)	
Pure fruit juices	0.074***	
	(0.029)	
Water	-0.768	-1.148
	(0.540)	(0.594)

^(a)^ Home Scan Panel (HSP)

^(b)^ Italian Household Expenditure Survey (HES)

Dependent variables are per capita purchased quantities.

Standard errors (in brackets) are clustered by household when using HSP data.

* p<0.1;

** p<0.05;

*** p<0.01.

Heavy purchasers are defined for each drink group as households whose per capita purchases laid within the top 25% in 2011.

## Robustness checks and additional results

### Difference-in-difference using water as the placebo good

The key identification assumption behind Eq (1) is that any selection bias implied by using data from Italy to build the counterfactual and not captured by the fixed effects is either constant over time, or—if it evolves over time—this evolution is linear. The relatively short time span covered by our home-scan data, the smaller size of the Italian HSP data-set and the potential measurement errors make it difficult to provide stringent tests of this assumption. We explore an alternative specification based on a different identification assumption for the policy effect, which does not require the use of the Italian data.

More specifically, mineral and spring water can be used as a “placebo” good under the assumption that the tax does not affect the price and purchases of mineral and spring water in France. Taken in its stricter interpretation, this assumption rules out substitution between taxed drinks and water. Under a more relaxed view, even in presence of cross-price responses and substitutions, one might refer to a different outcome definition, i.e. the price of drinks relative to water prices and the difference between drinks purchases and water purchases. The fact that average water prices and purchases have been stable in France between 2011 and 2012 (see Tables 2 and 3) suggests that the assumption behind this identification strategy is reasonable. Operationally, we proceed by defining our outcome measures as $Z_{ht}=Y_{ht}^{G}-Y_{ht}^{W}$ where $Y_{ht}^{G}$ is the the selected outcome for the *G*-th drink and $Y_{ht}^{W}$ is the corresponding outcome for water. Under this specification, the DID equation, which assumes parallel trends between the outcome on the selected drink and the corresponding outcome on water, is the following:

$$\begin{array}{c} Z_{ht}=\gamma_{h}+\zeta_{hs}+\delta P_{ht}+\eta_{ht} \end{array}$$ (2)

where *γ*_*h*_ are cross-sectional fixed effects; *ζ*_*hs*_ is a set of seasonal effects; *P*_*ht*_ is a binary variable equal to 1 for observations referring to the year 2012 when the tax is in place, and 0 otherwise; and *η*_*ht*_ is a randomly distributed error term. The coefficient *δ* corresponds to the DID interaction term and yields the average effect of the tax on exposed observations.

Table 8 reports the estimates from Eq (2) when prices are the outcome of interest.

**Table 8:** Robustness check: Tax impact on real prices, water as placebo good, France.

	Weekly regional prices	Monthly CPIs	
	2011-12	2007-16	2011-12
Soft drinks	0.057***	0.090***	0.088***
	(0.008)	(0.006)	(0.005)
Regular soft drinks	0.059***		
	(0.009)		
Diet soft drinks	0.068***		
	(0.009)		
Fruit Juices	0.026***	0.050***	0.046***
	(0.007)	(0.004)	(0.002)
Non-pure fruit juices	0.019***		
	(0.007)		
Pure fruit juices	0.023**		
	(0.009)		
N	104	120	24

Prices are real (deflated by the overall CPI) and in logs.

* p<0.1;

** p<0.05;

*** p<0.01.

The estimates of the tax effects are consistent with those of the DID Eq (1), and more efficient. Estimates are slightly larger than previous ones, as the increase in soft drink prices is estimated at +5.7% and +8.8% when using 2011-12 HSP weekly regional prices and CPI prices, respectively. As the sample is extended to include CPI data between 2007 and 2016, the estimated effect becomes larger (+9%), again validating results from the two-countries application. These estimates also confirm a slightly larger increase in the price of diet soft drinks compared to regular ones. A smaller tax effect, but still significant, is obtained for fruit juices, with a 2.6% price increase with HSP data, between +4.6% and +5% with CPI data, depending on the estimation window. As before, there is little difference between the price response of taxed non-pure juices and non-taxed pure juices, which may suggest that the tax has been diluted across the two categories.

Overall, the evidence that prices have responded to the tax seems quite strong. We also apply the model to purchase data, again with a distinction between the full sample and the sub-sample of heavy consumers. Results are shown in Table 9. Estimates from Eq (2) are similar in size to those from Eq (1), but standard errors are now smaller, and a small (-85 ml per capita per week) but significant effect is found for soft drinks, relatively larger for regular soft drinks (-58 ml per capita per week) compared to diet soft drinks (-28 ml per capita per week, non significant). When water is used as the placebo good, we also find a small reduction in purchases of fruit juices (-59 ml per capita per week), which is mainly driven by a significant reduction in non-pure fruit juices (-39 ml per capita per week).

**Table 9:** Robustness check: Tax impact on purchased quantities, water as placebo good, France.

	Full sample	Heavy purchasers
Soft drinks	-0.085*	-0.224**
	(0.046)	(0.099)
Regular soft drinks	-0.058**	-0.165***
	(0.024)	(0.050)
Diet soft drinks	-0.028	0.004
	(0.023)	(0.017)
Fruit Juices	-0.059	-0.145
	(0.045)	(0.103)
Non-pure fruit juices	-0.039*	-0.051
	(0.023)	(0.046)
Pure fruit juices	-0.020	-0.084
	(0.023)	(0.054)

Source: French and Italian home-scan data.

Dependent variables are per capita purchased quantities.

Standard errors (in brackets) are clustered by household.

* p<0.1;

** p<0.05;

*** p<0.01.

As we restrict the sample to heavy consumer, we find again larger impacts. The significant reduction in soft drink purchases is estimated at about 224 ml per capita per week, mainly because of reduced purchases of regular soft drinks (-165 ml). No significant effects emerge for fruit juices, but the sign is negative and the size effect (-145 ml) similar to the one obtained when using Italian HES data as the natural control group.

### Price pass-through: Time patterns

A further alternative model specification consists in a basic fixed time effect model, where the quarterly time effects are allowed to be different between the treated and the control groups over the whole time span. Since no explicit formulation of the time period when the policy is implemented enters the model, this specification allows to follow the evolution in the outcome difference between the two countries. A graphical inspection of the estimates of the differential time effect may be useful to identify: (a) whether the policy implementation is associated with a shift in the differential time effect; (b) potential time patterns in the effects of the policy. Thus, we estimate the following model:

$$\begin{array}{c} Y_{ht}=\gamma_{h}+\lambda_{0t}+\lambda_{1t}F_{ht}+\eta_{ht} \end{array}$$ (3)

where *Y*_*ht*_ is the outcome observed on cross-sectional unit *h* at time *t*; *γ*_*h*_ are cross-sectional fixed effects; λ_0*t*_ + λ_1*t*_ *F*_*ht*_ are time fixed effects, differently specified for French observations (*F*_*ht*_ = 1) and Italian observations (*F*_*ht*_ = 0); and *η*_*ht*_ is a randomly distributed error term. An analysis of the time patterns in λ_1*t*_ may provide insights on policy effects and their evolution over time.

**Fig 2:**
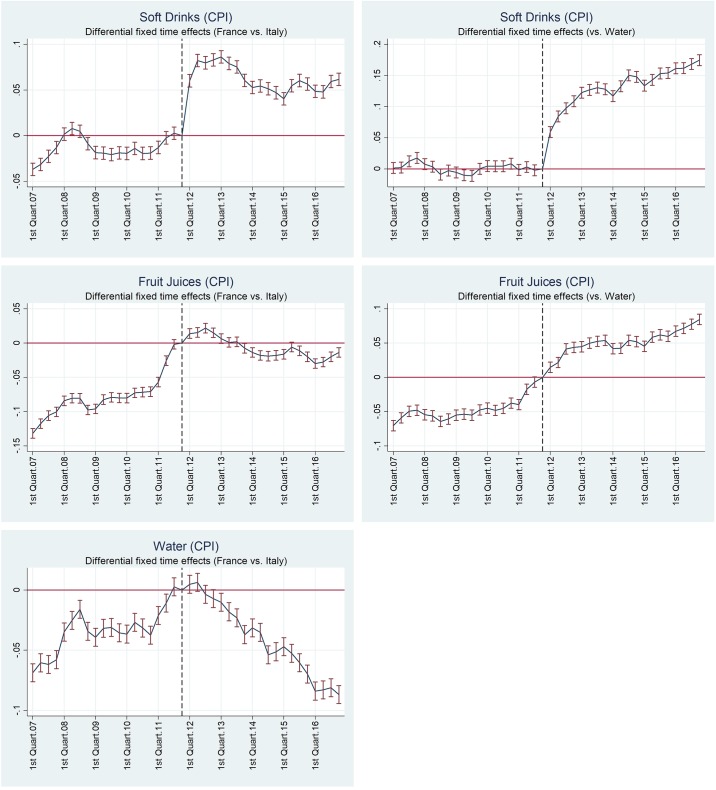
Differential fixed time effects in real consumer price indices, 2007-16. The graphs show the estimated differential fixed time effects for France relative to Italy (left graphs) and for each good relative to mineral and spring water CPIs (right graphs) according to Eqs (3) and (4), respectively. Time effects are relative to December 2011 = 0, bars show the 95% confidence intervals.

Similarly, one may look at the differential effect within the model based on French data only, using water as the counterfactual. The specification becomes:

$$\begin{array}{c} Z_{ht}=\gamma_{h}+\delta_{t}+\eta_{ht} \end{array}$$ (4)

where *Z*_*ht*_ is the same differential outcome variable as in Eq (2), and *δ*_*t*_ are fixed time effects which capture the evolution of the outcome variable relative to water as the counterfactual good. Fig 2 shows a graphical representation of the estimates of differential fixed time effects from Eqs (3) and (4) for CPIs over the 2007-2016 period.

Both specifications clearly identify the shift in real prices of soft drinks induced by the introduction of the tax in January 2012. Considering Italian consumer prices as the counterfactual, the peak impact is reached by the end of 2012, with a real increase around 8%, then it reduces to 5% in 2013 and is relatively stable thereafter. Estimates for the fruit juice aggregate confirm the smaller impact as captured by the DID models, and indicate a sharp increase before the introduction of the tax, then a further short-lived increase.

When the differential fixed time effects are estimated using French data only and water as the counterfactual good, the timing of the price increases are similar for both soft drinks and fruit juices. A sharper increase is observed in 2012, but there is no decline thereafter, as prices remain stable or they are even slightly increasing for soft drinks.

The Supporting Information S1 Fig reports results of the differential models applied to HSP regional weekly prices. The same exercise can be replicated for purchased quantities, but the limited time span of available data (8 quarters) and the lack of average significant and consistent impacts as detected by the previous difference-in-difference models generates findings of limited interest. Those estimates are nevertheless available from the authors on request.

## Conclusions

The ultimate impact of a soda tax is subject to many elements of uncertainty related to price transmission, firm strategic behaviors and consumer response and substitution patterns. The existing evidence on this type of measure rests primarily on simulations, but recently there have been several policies that could be evaluated after their implementation. One challenge in these ex post evaluations is the consideration of pre-existing trends and confounding effects, or simply the lack of an appropriate control group.

In our assessment of the 2012 French soda tax we address this challenge by referring to a natural control group for French households that were exposed to the introduction of a tax on sweetened soft drinks starting from January 2012. By looking at two regions in France and two neighbouring Italian regions across the border before and after the tax, we open the way to a DID estimation of the tax impact. As an alternative specification, we also estimate the tax effects using French data only, but considering water as the counterfactual good within each household participating in the panel. The availability of panel data on home purchases allows to control for household heterogeneity and non-linear time trends via a fixed effect specification. Under this specification, we have estimated the tax impact on market prices and purchased quantities.

We provide good evidence that the tax—which is applied to producers and importers—has been fully transmitted to consumer prices of soft drinks, while transmission for fruit juices has been partial, and may have been anticipated relative to the tax implementation date. The evidence on purchase responses is less clear and consistent across models, as the tax rate is too small to generate meaningful responses, especially in relation to measurement errors in the home scan data. The evidence of a reduction in purchases is very weak, and even considering the largest estimates, it suggests very small reduction in soft drink purchases (less than half a litre per capita per year). We find some evidence of a larger response by the sub-sample of heavy purchasers, with estimates that point out at reductions in soft drinks purchases between 6.8 and 11.4 litres per capita per year. Fruit juices and water do not seem to have been affected by the tax.

Suggestive evidence of the (over-)transmission of the French tax to the market prices had been provided by previous studies [18, 20] and our ex post evidence confirms these findings. In terms of changes in purchases induced by the tax, our estimates find at most a very small reduction for soft drinks, and slightly larger for those purchasing larger quantities. However, due to the low tax rate and because of the noise in the data, it is not possible to identify these effects with sufficient precision. Overall, a 10ml reduction in weekly purchases of soft drinks as captured by the double difference model means approximately a 3% reduction relative to 2011 average levels, and considering a 5% increase in price, this translates into an own-price elasticity of -0.60. When considering heavy purchasers, the average amount of purchased soft drinks over one year is about 108 litres, and the estimated reduction corresponds to about 10% of the pre-tax levels, hence an elasticity around 2 (or 1.2 when considering the CPI estimate of an 8.2% price increase). These elasticities seem reasonable, and the low response of purchases in line with the low tax rate. The detection of a higher response by heavy consumers may be explained in two ways. First, it is consistent with rational addiction theory [24], which predicts that even small changes in consumption have large effects on the budget of heavy consumers, hence they become more responsive to price changes. Second, if the tax is perceived to be a signal about potentially adverse health effects, it is reasonable that heavy consumers make larger adjustments.

Under a methodological point of view, the application of econometric techniques for quasi-experimental data provided robust and convincing results when working with prices, whether using CPIs or home scan data. Findings on HSP purchase data were less consistent and precise. This is not surprising, given that relatively low price changes induce small consumer responses, but we also found clear signs of measurement problems. Home scan data are a valuable source of information, but the noise in the data (especially due to zero observations and frequency of purchases) increases with the level of product detail, and is magnified when looking at smaller samples. Furthermore, the availability of relevant socio-demographic information was limited in our data-set, and additional covariates (e.g. income, better geocoding, education levels, etc.) would enable more powerful identification strategies. The quality of the data is variable across countries, mostly because of sample sizes. For example, the UK Kantar Worldpanel consists of more than 30,000 households, the Italian panel had less than 6,000 households in 2012 (now about 10,000).

Our current data do not allow inference on the longer term effects of the tax, and even with a longer time series it would become difficult to assume that the DID model can isolate the tax effect from other confounding factors intervening in the four regions. A further limitation in our study design must be acknowledged, as our data only cover drinks purchased for home consumption, but out-of-home consumption behaviour is likely to be very relevant to assess the ultimate weight or health impact of the tax. Still, our quasi-experimental setting can be compared to other recent ex post evaluations on similar fiscal measures in Mexico, Hungary and the city-level tax in Berkeley. Evaluations of these taxes found a complete pass-through and significant reduction in purchases which ranges between 7% and 20%. Our results are much less encouraging, but they are consistent with some price effect, and the idea that soda taxes larger than the French one may have a meaningful impact on purchases, especially for those groups with very high consumption levels.

## Supporting information

S1 Appendix Classification of households by socio-economic status. Harmonization between SES classification of the Italian and French home-scan panels.
(PDF)

S1 Fig Differential fixed time effects: Regional prices from home-scan data. Estimates refer to the differential fixed time effects for French regions relative to Italian regions (left graphs) and for each good relative to mineral and spring water average weekly purchase prices (right graphs) according to Eqs (3) and (4), respectively. The effects are relative to December 2011 = 0, bars show the 95% confidence intervals.
(TIF)

S1 Data Consumer price indices, nominal. The data-set contains the CPIs for soft drinks, fruit juice and water for France and Italy, over the period January 2007-December 2016.
(DTA)

S2 Data Consumer price indices, real. The data-set contains the CPIs for soft drinks, fruit juice and water for France and Italy, over the period January 2007-December 2016. Data are deflated using the all-item Consumer Price Index for each country.
(DTA)

S1 Syntax Stata code for harmonizing French and Italian home-scan panel data. The code contains the Stata commands to read the raw Italian and French data, and harmonize variables for subsequent analysis.
(DO) Click here for additional data file.

S2 Syntax Stata code to obtain estimates reported in the paper. The code contains the Stata commands to generate the estimates reported in Tables 3, 4, 5, 6, 7, 8 and 9, Fig 1 and S1 Fig.
(DO) Click here for additional data file.

## References

[1] Chriqui JF, Eidson SS, Chaloupka FJ. State Sales Taxes on Regular Soda (as of January 2014). Bridging the Gap Program, Health Policy Center, Institute for Health Research and Policy, University of Illinois at Chicago; 2014. Available from: www.bridgingthegapresearch.org.

[2] Paarlberg R, Micha R, Mozaffarian D. Can US Local Soda Taxes Continue to Spread? Food Policy. 2017;71:1–7. 10.1016/j.foodpol.2017.05.007 PMC8442984 34531634

[3] Guerrero-López CM, Unar-Munguía M, Colchero MA. Price elasticity of the demand for soft drinks, other sugar-sweetened beverages and energy dense food in Chile. BMC Public Health. 2017;17(1):180 10.1186/s12889-017-4098-x 28183287 PMC5301435

[4] Colchero MA, Rivera-Dommarco J, Popkin BM, Ng SW. In Mexico, Evidence Of Sustained Consumer Response Two Years After Implementing A Sugar-Sweetened Beverage Tax. Health Affairs. 2017;36(3):564–571. 10.1377/hlthaff.2016.1231 28228484 PMC5442881

[5] Briggs AD, Mytton OT, Kehlbacher A, Tiffin R, Rayner M, Scarborough P. Overall and income specific effect on prevalence of overweight and obesity of 20% sugar sweetened drink tax in UK: econometric and comparative risk assessment modelling study. British Medical Journal. 2013;347:f6189 10.1136/bmj.f6189 24179043 PMC3814405

[6] Mytton OT, Clarke D, Rayner M. Taxing unhealthy food and drinks to improve health. British Medical Journal. 2012;344:e2931 10.1136/bmj.e2931 22589522

[7] Fletcher JM, Frisvold D, Tefft N. Can soft drink taxes reduce population weight? Contemporary Economic Policy. 2010;28(1):23–35. 10.1111/j.1465-7287.2009.00182.x 20657817 PMC2908024

[8] WHO. Fiscal policies for diet and prevention of noncommunicable diseases: technical meeting report, 5-6 May 2015, Geneva, Switzerland. WHO; 2016.

[9] Cornelsen L, Mazzocchi M, Green R, Dangour AD, Smith RD. Estimating the Relationship between Food Prices and Food Consumption—Methods Matter. Applied Economic Perspectives and Policy. 2016;38(3):546–561. 10.1093/aepp/ppw010

[10] Redondo M, Hernandez-Aguado I, Lumbreras B. The impact of the tax on sweetened beverages: a systematic review. The American journal of clinical nutrition. 2018;108(3);548–563. 10.1093/ajcn/nqy135 30535085

[11] Colchero MA, Salgado JC, Unar-Munguía M, Molina M, Ng S, Rivera-Dommarco JA. Changes in prices after an excise tax to sweetened sugar beverages was implemented in Mexico: evidence from urban areas. PLoS ONE. 2015;10(12):e0144408 10.1371/journal.pone.0144408 26675166 PMC4682930

[12] Caro JC, Corvalan C, Reyes M, Silva A, Popkin B, Smith Taillie L. Chile’s 2014 sugar-sweetened beverage tax and changes in prices and purchases of sugarsweetened beverages: An observational study in an urban environment. PLoS medicine. 2018;15(7):e1002597 10.1371/journal.pmed.1002597 29969444 PMC6029755

[13] Nakamura R, Mirelman AJ, Cuadrado C, Silva-Illanes N, Dunstan J, Suhrcke M. Evaluating the 2014 sugar-sweetened beverage tax in Chile: an observational study in urban areas. PLoS medicine. 2018;15(7):e1002596 10.1371/journal.pmed.1002596 29969456 PMC6029775

[14] Falbe J, Thompson HR, Becker CM, Rojas N, McCulloch CE, Madsen KA. Impact of the Berkeley excise tax on sugar-sweetened beverage consumption. American Journal of Public Health. 2016;106(10):1865–1871. 10.2105/AJPH.2016.303362 27552267 PMC5024386

[15] Silver LD, Ng SW, Ryan-Ibarra S, Taillie LS, Induni M, Miles DR, et al Changes in prices, sales, consumer spending, and beverage consumption one year after a tax on sugar-sweetened beverages in Berkeley, California, US: a before-and-after study. PLoS Medicine. 2017;14(4):e1002283 10.1371/journal.pmed.1002283 28419108 PMC5395172

[16] Cawley J, Frisvold D, Hill A, Jones D. The Impact of the Philadelphia Beverage Tax on Purchases and Consumption by Adults and Children. National Bureau of Economic Research. 2018;No w25052. 10.1016/j.jhealeco.2019.102225 31476602

[17] Roberto CA, Lawman HG, LeVasseur MT, Mitra N, Peterhans A, Herring B, Bleich SN. Association of a beverage tax on sugar-sweetened and artificially sweetened beverages with changes in beverage prices and sales at chain retailers in a large urban setting. Journal of the American Medical Association. 2019;321(18):1799–1810. 10.1001/jama.2019.4249 31087022 PMC6518342

[18] Berardi N, Sevestre P, Tépaut M, Vigneron A. The impact of a ‘soda tax’ on prices: evidence from French micro data. Applied Economics. 2016;48(41):3976–3994. 10.1080/00036846.2016.1150946

[19] Etilé F, Lecocq S, Boizot-Szantai C. The Incidence of Soft-Drink Taxes on Consumer Prices and Welfare: Evidence from the French Soda Tax; 2018.

[20] Bonnet C, Réquillart V. Impact of cost shocks on consumer prices in vertically-related markets: the case of the French soft drink market. American Journal of Agricultural Economics. 2013;95(5):1088–1108. 10.1093/ajae/aat055

[21] USDA Foreign Agricultural Service. France to tax soft drinks—U.S. Companies to pay the most. GAIN Report (Global Agricultural Information Network); 2011. FR9077.

[22] Deaton A. Quality, quantity, and spatial variation of price. The American Economic Review. 1988;78(3):418–430.

[23] Crawford I, Laisney F, Preston I. Estimation of household demand systems with theoretically compatible Engel curves and unit value specifications. Journal of Econometrics. 2003;114(2):221–241. 10.1016/S0304-4076(03)00083-6

[24] Becker GS, Murphy KM. A theory of rational addiction. Journal of Political Economy. 1988;96(4):675–700. 10.1086/261558

