# Supplementary material for: The impact of the French soda tax on prices and purchases. An ex post evaluation
Source: PLoS One. 2019 Oct 11;14(10):e0223196. doi: 10.1371/journal.pone.0223196 (PMC6788734; doi:10.1371/journal.pone.0223196)
Supplement: S1 Appendix — Harmonization between SES classification of the Italian and French home-scan panels. (PDF) [file pone.0223196.s001.pdf]

S1 - Appendix

Classification of households by socio-economic status

Information on household income is only available for the French dataset. The Italian dataset provides a five-classes classification of households based on a scoring system on socio-economic status, derived from information on home property, possession of durable goods, education level, car ownership and job position. Italian households were classified into the five classes depending on their ranking on the score as follows: (1) top 15%; (2) 65th-85th percentile; (3) 35th-65th percentile; (4) 15th-35th percentile; (5) bottom 15%. We applied the same classification rule to French households (prior to extraction of the regional sub-sets) based on household income.
