# Supplementary figures and images for: The impact of the French soda tax on prices and purchases. An ex post evaluation
Source: PLoS One. 2019 Oct 11;14(10):e0223196. doi: 10.1371/journal.pone.0223196 (PMC6788734; doi:10.1371/journal.pone.0223196)

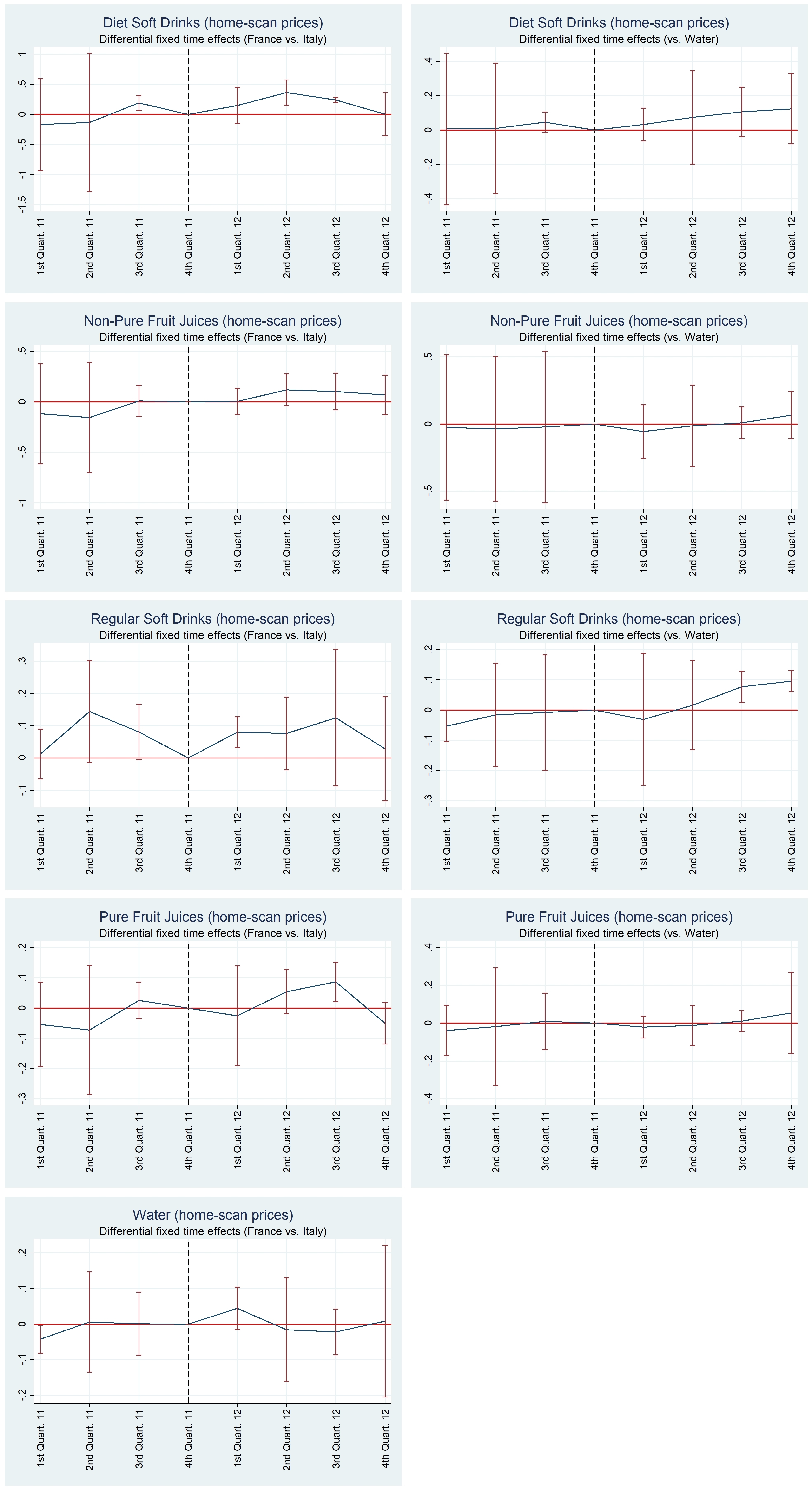

Supplement: S1 Fig — Estimates refer to the differential fixed time effects for French regions relative to Italian regions (left graphs) and for each good relative to mineral and spring water average weekly purchase prices (right graphs) according to Eqs (3) and (4), respectively. The effects are relative to December 2011 = 0, bars show the 95% confidence intervals. (TIF) [file pone.0223196.s002.tif]
